# Is Redshirting Beneficial for Reading Acquisition Success?

**DOI:** 10.3389/fpsyg.2020.01165

**Published:** 2020-06-03

**Authors:** Ana Sucena, Cátia Marques, Ana Filipa Silva, Cristina Garrido, Rui Pimenta

**Affiliations:** Research and Intervention Reading Centre, Polytechnic Institute of Porto, Porto, Portugal

**Keywords:** redshirting, reading acquisition, phonemic awareness, letter-sound knowledge, reading precursors

## Abstract

The controversy around the effect of academic redshirting on reading acquisition continues receiving attention in the international literature. However, few studies are known with non-English speaking children. In this study we intend to understand this phenomenon with 698 Portuguese speaking first graders, 360 girls (51.6%), aged between 5 years old and 8 months and 7 years old and 6 months (*M* = 6.3 months, *SD* = 3.9 months). Reading acquisition precursors were assessed namely phonemic awareness and letter-sound knowledge. Results reveal that 5.9% of first graders are redshirted. Clusters analysis indicated two clusters per variable. Cluster 1 with low phonemic awareness and letter-sound knowledge and low socioeconomic status, cluster 2 with high phonemic awareness and letter-sound knowledge and medium-high socioeconomic status. The cluster results suggest a prevalence of 24.5% children at risk of having learning difficulties. The MANOVA indicated that only socioeconomic status has an effect on phonemic awareness and letter-sound knowledge, with children from medium-high level presenting higher results. It is concluded that redshirting did not bring additional advantages for reading acquisition success. Implications about the importance of education in order to lessen those differences, as well as prevent difficulties are presented.

## Introduction

Redshirting or holding age-eligible children back for a year prior to their enroling in kindergarten (Frey, 2005) is a borrowed term from sports academic literature that refers to the period of time when an athlete forgo a year of competition, in order to develop skills and gain maturity (e.g., Graue and Diperna, 2000; Range et al., 2011). The application of this assumption to kindergarten students ensures that such students are older than their same grade peers, thus they are part of the oldest in their class and remain so throughout their school years, enjoying the presumed advantages of age (“the gift of time”) (Noel and Newman, 2008). In Europe (e.g., France, Portugal) as well as China and Canada (the French part), children start their formal education at the age of 6 years old in primary education schools. Before that, there is a preschool system that is facultative, once it is believed that the family has an essential role in the preschool education (Direção-Geral da Educação, 2016). However, in Portugal, there is no specific curriculum, for children in the pre-educational system. Instead, there are curriculum guidelines that orient educators in their practices (Direção-Geral da Educação, 2016). In this case, redshirted students stay in the same class as younger children with the same guidelines.

Regardless of the type of educational system, the controversy around the practice of academic redshirting continues to receive attention in the popular press (e.g., Lin et al., 2009; Safer, 2012; Moyer, 2013; Ashbrook, 2014; Schanzenbach and Larson, 2017) and in the academic field (e.g., Mendez et al., 2014; Huang, 2015; Barnadr-Brak and Albright, 2017; Fortner and Jenkins, 2018). A few studies have demonstrated some short-term benefits of academic redshirting (e.g., Datar, 2006; Bedard and Dhuey, 2006; Pong, 2009), although the majority of studies have shown no particular lasting advantages for redshirting students (e.g., Graue and Diperna, 2000; Gladwell, 2008; Cascio and Schanzenbach, 2016; Gottfried et al., 2016; Attar and Cohen-Zada, 2018).

One of the most commonly mentioned benefits of redshirting is to rely on more sophisticated cognitive development as a result of higher age (e.g., Datar, 2006; NICHD Early Childhood Care Research, 2007; Dougan and Pijanowski, 2011; Huang, 2015). However, there is some controversy regarding these premise (Attar and Cohen-Zada, 2018). Some studies emphasise the importance of variables other than age to explain cognitive differences, namely, the child’s sex, the family’s economic background, and parent’s higher education (Attar and Cohen-Zada, 2018). Other mentioned benefits of being redshirted is that children are less likely to be retained (e.g., Datar, 2006; Huang, 2014), and less likely to be diagnosed with learning disabilities (Peterson et al., 2010; Huang, 2015). To those who were already diagnosed with learning disabilities, redshirting does not appear to be especially beneficial (Barnard-Brak et al., 2015). Some studies have also found that the youngest children in a classroom are at an elevated risk of being diagnosed with Attention Deficit Hyperactivity Disorder (Whitely et al., 2019). Older students are considered easier to teach, and teachers can implement more rigorous teaching methods with those students (Dougan and Pijanowski, 2011; Range et al., 2011). Some parents’ state that being older helped their child develop better friendships with other children (Dougan, 2015). However, Range et al. (2011), suggest that academic redshirting has a negative outcome on social-emotional aspects. Their research concludes that being older than their classmates can increase behaviour problems and negatively impacts on self-esteem.

The literature also refers some disadvantages of being redshirted. Researchers have found that redshirted students, compared to on-time students, had a higher probability of being placed in a special education program (Graue and Diperna, 2000; Mendez et al., 2014). The level of demotivation of these children, as a result of lack of stimulation, is another mentioned disadvantage (Schanzenbach and Larson, 2017). Redshirted children are associated with higher developed behavioural problems and substance abuse (Byrd et al., 1997; Guagliardo et al., 1998), social problems (e.g., troubles to make friends), lower earnings as adults (e.g., Deming and Dynarski, 2008), lower homework completion rates, and lower academic performance levels (Martin, 2009). Another disadvantage of redshirting is the associated increase in the academic demands in kindergarten, resulting in curriculum escalation (Huang, 2015). This curriculum escalation makes parents and teachers fearful that young 5-year-olds will fail in their first grade and consider keeping them one more year in kindergarten (Graue and Diperna, 2000). Keeping older and high-achieving children in kindergarten will contribute to this curriculum escalation.

To sum up, there is no congruence in the literature regarding the practice of redshirting. Accordingly, the assumption that children benefitting from being in a class with well-behaved and high-achieving children in order to feel challenged should weigh more heavily for parents’ and educators’ when faced with the decision regarding postponing the entry in 1st grade.

The concerns that lead parents, teachers, or school administrators to suggest redshirting are most often related to the child’s physical, social, and emotional maturity, as well as to the child’s birthday, if it is late in the year (Range et al., 2011; Schanzenbach and Larson, 2017; Attar and Cohen-Zada, 2018). The decision as to whether they should enroll their child in kindergarten, when he/she is first eligible or holding him/her back for a year is typically hard for parents, who have the last word regarding the decision on sending their children to school when they are first age-eligible. This choice has substantially different perspectives concerning their decision-making, as well as different preparation activities for the holdout year. Some parents base their decisions by valuing the benefits of time on reading and spelling learnings for example, others are motivated by a philosophy that give them a more active role in shaping their child’s experiences during that year (Noel and Newman, 2008).

The reading and spelling learnings are based on the learning rhythm, which is associated with the characteristics of the orthography in which the child acquires reading. For English speakers, the orthography has more than 44 phonemes (with more than 1100 possible pronunciations) and 229 graphemes (Rao, 2018). For those, in turn, more initial difficulties with reading are presented than for Spanish-speaking (24 phonemes for 30 graphemes in Spanish) or Portuguese speaking children (35 phonemes for 67 graphemes in European Portuguese) for example (Serrano et al., 2011). This is related to the orthographic depth. The less transparent an orthography is, the greater the likelihood for children to experience difficulties, especially in the initial phase of reading acquisition. Spanish is considered a very transparent orthography (Clinton et al., 2013), European Portuguese is considered relatively transparent, thus allowing children to develop grapheme-phoneme correspondences without significant difficulties (Sucena et al., 2009; Ziegler et al., 2010). Even if Portuguese speakers can experience fewer difficulties than English speakers, redshirting also has practical significance to diverse stakeholders in the Portuguese context.

The question that arises and that is not clear in the literature is whether age is a variable that we worriedly should take into consideration when deciding whether children should enter school or not. This is the question we aim to clarify with our study. Recent Portuguese data (Conselho Nacional de Educação, 2018, regarding 2016/2017 data) reveals that 89.8% of 1st graders were 6 years old, 9.3% were 7 years old, and only 0.24% were 5 years old. Regarding the last year of preschool, it should be noted that only 4% of children enroled in preschool were 6 years old in 2016/2017. Between 2007/2008 and 2016/17, the percentage of 6 years old students in the preschool years increased from 1 to 4%. Between 2010/11 (1.9%) and 2011/12 (1.8%) it decreased slightly, in 2012/13 (2.3%) it rose, and in 2013/14 (2.2%) it slightly decreased again. Between 2014/15 (2.6%), 2015/16 (2.9%), and 2016/17 (3.9%) the growth was gradual. In 2016/2017, differences between sexes were significant, in result of a tendency for boys to spend more time at this educational level than girls (Conselho Nacional de Educação, 2018).

International data shows that redshirted students are most likely Caucasian boys (Attar and Cohen-Zada, 2018), from a non-minority community (Lenard and Peña, 2018), from higher income families (Frey, 2005) and have late summer birthdays (Range et al., 2011; Huang, 2014; Schanzenbach and Larson, 2017). Research also suggests that the variation in redshirting rates may be caused by sample and region (Huang, 2015; Attar and Cohen-Zada, 2018), but most studies are focused on North American samples. In the United States of America, redshirting has been declining over time (2010–2013) (Huang, 2015). In Portugal, as far as the authors’ knowledge goes, the information is scarce, vague, and recent (Direção-Geral da Educação, 2016). Studies using samples from different countries are necessary for a more though understanding of the redshirting phenomena.

In this study, we intend to characterise the prevalence of redshirting in Portuguese first graders (for which the age was adopted as the key variable, as redshirted children are those who are 7 years old); to characterise first-graders regarding sociodemographic variables such as sex, age, and Socioeconomic Status (SES), as well as regarding reading success precursors – Phonemic Awareness (PA) and Letter-Sound Knowledge (LSK). PA and LSK are two fundamental basic skills to acquire reading (Kyle et al., 2013). The assessment of these skills was based on the fact that these are the most robust precursors for reading acquisition success, thus being adopted for early identification of children at risk for developing learning difficulties (Lyytinen et al., 2012; Hulme and Snowling, 2015; González et al., 2017). There is a relationship between PA, LSK and families’ SES, as higher SES and parental education are protecting factors for successful reading and spelling acquisition (Zalewska-Łunkiewicz et al., 2016). We intend to analyse whether there is a variation in these skills that accompanies age – and in this way to reflect upon the impact of redshirting on reading acquisition success. In order to isolate what the literature indicates as confounding variables, sex and SES were analysed in parallel with age. Specifically, we intend to understand whether the prevalence of poor PA and LSK at the onset of first grade is more prevalent across sex, age (5, 6, or 7 years old) and/or SES (Medium-High and Medium-Low). Based on the literature, we expect to find that Medium-High SES boys are more redshirted than Medium-High girls are. Once PA and LSK are the most robust precursors for reading acquisition success, and the main reason for redshirting is the presumed advantages of age, we expect older children would present better results on these skills than their peers, as well as the opposite – younger children (5 years old) would present the poorest results. Finally, it is our aim to define a cut-off that will contribute to the early screening of at risk children, as to allow early intervention. Recent data suggests that 20% of children at the beginning of the elementary school are at risk for developing reading acquisition difficulties (Scarborough et al., 2009; Vale et al., 2011). In order to give those children a fair chance the interventions should occur as early and effectively as possible (Ozernov-Palchik and Gaab, 2016; Porta and Ramirez, 2019).

## Materials and Methods

### Participants

We assessed 698 Portuguese first graders, 360 girls (51.6%) and 338 (48.4%) boys, aged between 5 years old and 8 months and 7 years old and 6 months (*M* = 6.3 months, *SD* = 3.9 months), attending 28 elementary education schools. From these, 15 (2%) come from a minority ethnic group (gypsies), and 33 (5%) have double nationality (they were born in Portugal but their mother or father is foreign). From those with double nationality, it is important to emphasise that for 13 (2%) of them, the native language is still Portuguese.

Observing Table 1, we can see that 503 were 6 years old, 154 were 5 years old, and 41 were 7 years old. Across 7 year old children there is a similar number of girls (*n* = 20) and boys (*n* = 21). The number of 7 years old is lower than 5 years old and 6 years old. Participants were classified according to their socioeconomic background, 385 (55.2%) students from medium-high SES and 313 students (44.8%) from medium-low SES. Across 7 years old medium-low SES represents roughly half of the children, when compared to medium-high SES (respectively, *n* = 14 and *n* = 27). The same tendency is observed with 5 years old, as medium-low SES represents roughly a quarter, when compared to medium-high SES (respectively, *n* = 66 and *n* = 88).

**TABLE 1:** Distribution of students by sex, age, and SEL.

	**Medium-high socioeconomic status**			**Medium-low socioeconomic status**			**Total (*n*)**
			
	**Girls (*n*)**	**Boys (*n*)**	**Total (*n*)**	**Girls (*n*)**	**Boys (*n*)**	**Total (*n*)**	
5 years old	43	45	88	36	30	66	154 22.1%
6 years old	133	137	270	106	127	233	503 72.1%
7 years old	11	16	27	9	5	14	41 5.9%
Total	187	198	385 55.2%	151	162	313 44.8%	698

### Instruments

Participants answered sociodemographic questions (e.g., sex, age, school, and school grade). PA and LSK were assessed using the European Portuguese Reading Assessment Battery (ALEPE, Sucena and Castro, 2011). PA was evaluated through the Phonemic Metalinguistic Awareness subtest. The letter-sound knowledge was evaluated through the subtests Letter-Sound and Letter Spelling. The mean of the two subtests was computed in order to create a final letter-sound knowledge variable.

The Phonemic Metalinguistic Awareness subtest intends to evaluate the child’s ability to identify the common phoneme in a couple of words. This subtest consists of three training items and twelve experimental items. Each item is formed by a pair of two-syllable words with simple and complex syllabic structures {CV [Consonant-Vowel (open syllable)] and CVC [Consonant-Vowel-Consonant (closed syllable)]}. The experimenter presents each pair of words orally and the child’s task consists on orally identifying the common phoneme. The result is the total of correct answers.

The Letter-Sound subtest aims to evaluate the child’s knowledge of letters. This test consists of reading a list of letters (lower case) displayed on the computer screen. This subtest consists of two training items and twenty-three experimental items. Both the sound and the letter name are accepted as correct answers. The result is the total number of letters correctly read.

The Letter Spelling subtest aims to evaluate the child’s knowledge of letters. It consists on writing letters dictated by the examiner (both lowercase and uppercase as well as print and cursive handwriting). This subtest consists of two training items and twenty-three experimental items. The result is the total number of letters correctly written by the child.

### Procedures

Participants were first graders attending 28 schools in a municipality in the north of Portugal. Authorisations were obtained from the school boards and those formally responsible for the child’s education (e.g., parents or other relatives), ensuring the voluntary participation of all children. The data collection took place at the beginning of the 2018/2019 school year, in a calm and quiet classroom context. Participants were evaluated through the ALEPE Assessment Battery individually, by speech therapists, teachers and psychologists with specific training in the test. Confidentiality of the data and results in the study were ensured. All recommended ethical care rules for this type of study were followed (e.g., informed consent, privacy and confidentiality) (Regulation No. 258/2011, Code of Ethics of the Order of Portuguese Psychologists). The participants were assessed at the beginning of the school year (during September and October 2018).

Statistical analyses were run with the Statistical Package for the Social Sciences (SPSS IBM) for Windows, version 25.0. Descriptive statistics (mean and standard deviation) were computed in order to describe the participants and the prevalence of redshirting in Portuguese first graders adopting age variable (between 5 and 7 years old). Then, a cluster analysis was conducted in order to group the participants by their level of PA and LSK. With the aim of confirming the accuracy of the clusters cut-off, a common standard cut off (Tomblin et al., 2003; Boyle et al., 2007) was computed for PA and LSK, using the mean value subtracting a standard deviation as criterion. Lastly, a multivariate analysis of variance (MANOVA) was conducted to verify the impact of sex, age, and SES on PA and LSK, and in this way to reflect upon the impact of redshirting on reading acquisition success. The SES was evaluated by the type of school context the students attend (TEIP or non-TEIP). TEIP is a Portuguese acronym, which means “Program of Educational Territories of Priority Intervention.” It is a governmental initiative, currently implemented in schools located in economically and socially disadvantaged territories, marked by poverty and social exclusion, where violence, indiscipline, neglect and school failure are manifested (Conselho Nacional de Educação, 2018). The MANOVA assumptions were analysed (multivariate normality violation, homogeneity of variance-covariance matrices, and sphericity). Since not all the MANOVA assumptions were fulfiled, the value of Pillai’s Trace was considered for the multivariate results (Tabachnick and Fidell, 2013).

## Results

The cluster analysis to group the participants into homogeneous groups according to their levels of PA and LSK indicates two clusters per variable. The frequency and percentage of participants per cluster are described in Table 2. The majority of participants was grouped in the medium high results cluster for both PA and LSK, with results around 50%. Participants with medium low results represent 1/5 for PA and 1/3 for LSK with mean results, respectively, of 0 and 3.

**TABLE 2:** Frequency and percentage of the participants per cluster (*N* = 698).

	**Clusters**	**N**	**%**	***M***	***SD***	**Minimum**	**Maximum**
Phonemic awareness^*a*^	M-Low PA	105	15%	0	0	0	0
	M-High PA	593	85%	6.86	3.69	3.17	10.55
Letter-sound knowledge^*b*^	M-Low LSK	235	33.7%	3.23	1.31	1.92	4.54
	M-High LSK	463	66.3%	11.99	5.18	6.81	17.17

**PA, Phonemic Awareness; LSK, Letter-Sound Knowledge; M, Mean; SD, Standard Deviation. ^*a*^Maximum correct answer 12. ^*b*^Maximum correct answer 23.**

In order to have a more thorough characterisation for each cluster, we further analysed the sociodemographic characteristics. Table 3 presents the characteristics for each cluster by sex, age, and SES.

**TABLE 3:** Frequency of participants per cluster according to sex, age and SES (*N* = 698).

**Clusters**	**Sex**		**Age**						**SES**	
	**Girls**	**Boys**	**5**	**%**	**6**	**%**	**7**	**%**	**Medium High**	**Medium Low**

**Phonemic awareness**										
1 Low PA	50	55	29	18.8	70	13.9	6	14.6	45	60
2 High PA	305	288	125	81.2	433	86.1	35	85.4	340	253

**Letter-sound knowledge**										

1 Low LSK	120	115	56	36.4	167	33.2	12	29.3	95	140
2 High LSK	218	245	98	63.6	336	66.8	29	70.7	290	173

Cluster 1 – low PA – is characterised mainly by boys, 6 years old and low average SES. There seems not to exist a prevalence tendency according to age in this cluster (respectively, 18.8, 13.9, and 14.6% for 5, 6, and 7 years old).

Cluster 2 – high PA – is characterised mainly by girls, 6 years old, from average medium-high SES. In the same way as for cluster 1, the percentage of 5 (81.2%), 6 (86.1%), or 7 (85.4%) years old in this cluster does not seem to variate considerably.

Concerning clusters related to LSK, cluster 1 – low LSK – is characterised mainly by girls, 6 years old, from medium low SES. The percentage of 5 (36.4%), 6 (33.2%), or 7 (29.3%) years old children in this cluster does not seem to variate considerably.

Cluster 2 – high LSK – is characterised mainly by boys, 6 years old, from medium-high SES. Once again, there does not seem to prevail any prevalence according to age (respectively, 63.6, 66.8, and 70.7% for 5, 6, and 7 years old).

Summing up, the cluster analysis indicate two groups that consistently vary according to the SES. Both sex and age do not seem to variate consistently. Regarding sex the low PA cluster is characterised by more boys than girls, whereas the low LSK cluster is characterised by more girls than boys (and the exact opposite tendency for high clusters). When analysing the sum of girls and boys in both low clusters, the exact same number (*n* = 170) is observed (respectively, 50 low PA + 120 low LSK girls, and 55 low PA + 115 low LSK boys). Regarding age the low PA cluster is characterised by more 5 years old, followed by 7 years old and finally 6 years old (respectively, 18.8, 14.6, and 13.9%) whereas the low LSK is characterised by more 5 years old, followed by 6 years old and finally 7 years old (respectively, 36.4, 33.2, and 29.3%).

The results of the cluster analysis suggest a prevalence of 24.5% at risk children, as computed from the average of 15% low PA and 33.7% LSK. In order to confirm the accuracy of the cluster analysis we computed a unanimously accepted cut-off for PA and LSK – subtracting 1.5 Standard Deviation to the mean. By adopting the 1.5 *SD* cut off 26.8% of the participants are identified as at risk for reading acquisition difficulties (187 participants).

The results of the MANOVA indicate a multivariate significant statistical effect of the SES Pillai’s Trace = 0.978, *F*(2, 685) = 7.701, *p* < 0.001, on both PA and LSK (Table 4).

**TABLE 4:** Effects of sex, age, and SES on phonemic awareness and letter-sound knowledge.

	**Sex**		**Age**					**SES**		
	**Girls**	**Boys**	***F*(1, 686)**	**5 years**	**6 years**	**7 years**	***F*(2, 686)**	**Medium high**	**Medium low**	***F*(1, 686)**

	(*n* = 338)	(*n* = 360)		(*n* = 159)	(*n* = 503)	(*n* = 41)		(*n* = 385)	(*n* = 385)	

	**Mean (*SD*)**	**Mean (*SD*)**		**Mean (*SD*)**	**Mean (*SD*)**	**Mean (*SD*)**		**Mean (*DP*)**	**Mean (*SD*)**	
PA	5.75 (4.02)	5.91 (4.36)	0.67	5.11 (4.03)	5.97 (4.21)	6.78 (4.37)	2.54	6.08 (4.17)	5.52 (4.22)	2.17
LSK	8.66 (5.59)	9.40 (6.27)	0.01	8.03 (5.42)	9.18 (5.96)	11.67 (7.22)	3.60	10.30 (6.17)	7.49 (5.30)	14.19***

***p < 0.001.

Univariate tests indicate a statistically significant effect of SES on letter-sound knowledge, *F*(1, 686) = 14.19, *p* < 0.001, with medium-high SES children presenting significantly better results than medium-low SES children.

## Discussion

The aim of this study was to analyse whether our data supports the benefits of redshirting. For that propose, we analysed the effect of age, sex and SES on PA and LSK. Specifically, this study intended to (1) define the prevalence of redshirting across Portuguese first-graders (2) characterise first graders regarding sex, age, SES and reading precursors, (3) analyse whether age, sex, and/or SES impact on the reading precursors – PA and LSK. Lastly, we defined a cut-off that will contribute to the early screening of at risk children.

Unsurprisingly, results reveal a higher prevalence of 6 years old, followed by 5 and finally by 7 years old children. This tendency is congruent to that described in other studies with Portuguese data (Conselho Nacional de Educação, 2018), which precisely state that the number of redshirted students is lower in comparison to the number of 6 years old.

We found a redshirting prevalence of 5.9%, without variation across sexes. The absence of differences across sex does not go in the same direction as the literature (international and national, respectively Attar and Cohen-Zada, 2018; Conselho Nacional de Educação, 2018) that state that the redshirted students are mainly boys. Our results indeed suggest that low performance on reading precursors does not vary with sex, as the number of boys and girls, despite age, is precisely the same. Regarding SES, the number of redshirted students with medium-low SES is lower than that observed across medium-high SES, despite the age or sex. These results are congruent with the literature that describes redshirting as more common in high SES families than within low SES families (e.g., Frey, 2005).

In order to characterise first graders performance on reading precursors we ran a cluster analysis to group the students according to sex, age and SES. The cluster analysis revealed two groups: medium low with poor results for both PA and LSK, along with medium low SES; and medium high with results around 50% for both PA and LSK. Similar results are presented in other studies highlighting the fact that higher family’ SES is a reading and writing predictor of success (Zalewska-Łunkiewicz et al., 2016).

We intended to verify if there is, in fact, an effect of sex, age and SES on PA and LSK. Results indicate SES as the only factor that explains the differences in PA and LSK. These results do not support the importance of sex or age as much as it supports the importance of SES. Being a girl or a boy or young or old does not seem to affect the performance on reading precursors. In this way, we can assume that children benefit from being in a class with high-achieving children in order to feel challenged and progress in their school learning. Based on the results of this study, we can conclude that redshirting did not introduce additional advantages for reading acquisition success, once among 5 years old (those who could have been redshirted), the percentage of poor results regarding reading precursors is similar to that found across 6 and 7 years old. Based on the literature (e.g., Frey, 2005; Range et al., 2011; Huang, 2014) we could suppose that among the 5 year old children with low SES the risk factor would be increased. However, the results of this study do not confirm that assumption as shown by the absence of an interaction between age and SES in the MANOVA. Only SES is associated with the precursors of successful reading acquisition. Once the SES appears as an important variable to explain the differences between first graders on PA and LSK, confirming what other studies have already found, we believe that this study can contribute to underline the importance of education in order to strike those differences. Public school should be an instrument that allow students who come from a disadvantaged socioeconomic environment to have opportunities to enrich their lives through educational support. The effect of SES should receive special attention with diagnosis assessments and early interventions in order to counteract the risk trend.

With the aim of defining a cut-off that will contribute to the early screening of at risk children, as well as to analyse the accuracy of the cluster analysis, that indicated 24.5% of at risk children, a 1.5 SD cut-off was computed regarding PA and LSK. The 1.5 SD cut off indicates a similar percentage of at risk children thus validating that 25% of beginning 1st graders are at risk. This percentage meets what is typically the statistic of children at risk in Portugal (Vale et al., 2011) and worldwide (Scarborough et al., 2009). This cut-off can help the practitioners to early identify and develop extra specific programs for those children. Our results also suggest there could be benefits from an early cut-off assessment, as an appropriate response to increase the quality of early education, adapting the academic curriculum to the developmental levels of the children, rather than raising the starting age of reading and writing acquisition and waiting for the presumed advantages of age (Fortner and Jenkins, 2017). The existence of a cut-off for signalling children at risk, regardless of other characteristics is of crucial importance, thus allowing, since the beginning of the first grade, the planning and implementation of reading promotion intervention.

In future studies it would be important to deepen the variability of PA and LSK dimensions regarding sex, age, and SES with larger groups of children and from different regions of the country. The use of other evaluation measures such as the level of parental education, frequency of preschool education and other developmental issues that can affect children’ performance could enable a more precise and detailed characterisation of the development and needs of these children. Congener’s studies are relevant to inform about reading and writing interventions, in the school context and to support their relevance to different contexts.

## Data Availability Statement

All datasets generated for this study are included in the article/supplementary material.

## Ethics Statement

Ethical review and approval was not required for the study on human participants in accordance with the local legislation and institutional requirements. Written informed consent to participate in this study was provided by the participants’ legal guardian/next of kin.

## Author Contributions

AS designed and supervised all the study. CM analysed the data and wrote the manuscript. AFS and CG collected and insert the data on SPSS. RP supervised the data analysis and revised the manuscript critically.

## Conflict of Interest

The authors declare that the research was conducted in the absence of any commercial or financial relationships that could be construed as a potential conflict of interest.
